# Calcinosis Cutis Inducing "Syringomatoid" Eccrine Hyperplasia: An Extremely Rare Example

**DOI:** 10.7759/cureus.66016

**Published:** 2024-08-02

**Authors:** Umar A Hussain, Audrey E Ahuero, Jamie A Tschen

**Affiliations:** 1 Histopathology, Manchester University NHS Foundation Trust, Manchester, GBR; 2 Ophthalmology, Ophthalmic Plastic Surgeons of Texas, Houston, USA; 3 Dermatopathology, St. Joseph Hospital, Houston, USA

**Keywords:** hyperplasia, eccrine duct, calcinosis, calcinosis cutis, eccrine hyperplasia, syringoma, syringomatoid

## Abstract

Reactive “syringomatoid” eccrine proliferations are a well-established phenomenon, which can show similar but less extensive histological features of a syringoma. The cut-off between syringomatoid hyperplasia and syringomas is subjective and given the considerable morphological overlap, it is possible they represent two points on the same spectrum. Syringomatoid hyperplasia has been associated with several conditions including neoplasms and inflammatory dermatoses. Herein, we describe an extremely rare case of syringomatoid hyperplasia occurring with calcinosis cutis in a 54-year-old Caucasian male. To the best of the authors’ knowledge, this is the first such case described in the literature.

## Introduction

Syringomas are well-recognized benign skin adnexal tumours that typically present as small flesh-coloured papules on the eyelids [1,2]. Histologically, they show “comma-shaped” or “tadpole” eccrine ductular nests typically confined to the superficial dermis - features that are so well recognised that they have almost become synonymous with syringoma [2]. However, hyperplastic eccrine proliferations producing similar “syringomatoid” ducts have long been recognized in the literature and have been described in association with inducing pathologies, such as neoplasms or inflammatory dermatoses [3]. We will be using the term “syringomatoid hyperplasia” to describe this phenomenon, but other terms such as “syringomatous dermatitis” have also been suggested [4]. Herein, we describe an extremely rare case of syringomatoid hyperplasia occurring with calcinosis cutis in a 54-year-old Caucasian male.

## Case presentation

**Figure 1:**
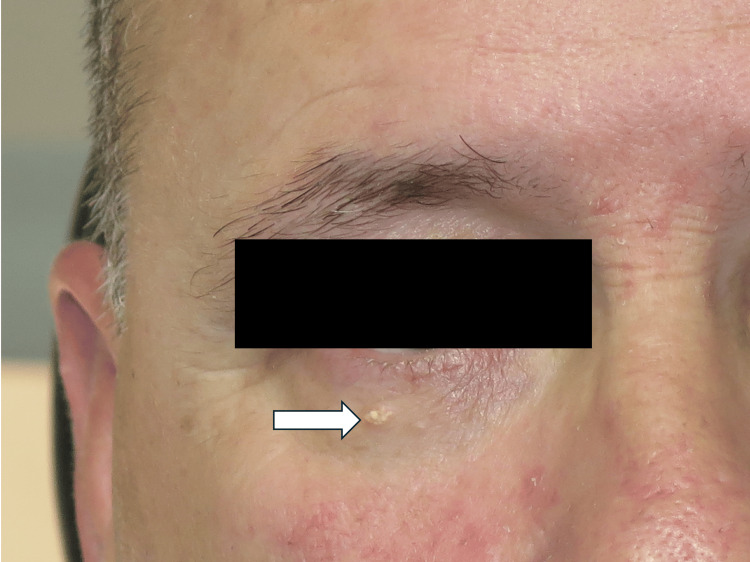
An isolated 4mm papule present on the right lower eyelid consisting of multiple white micronodules (arrow). The patient also shows features of rosacea.

A 54-year-old Caucasian male presented to the ophthalmologist with a one-year history of a lesion on the right lower eyelid. There were no symptoms related to the lesion except for a slight increase in size. He had a past medical history of hypothyroidism, gastroesophageal reflux disease, sleep apnea and arthritis. Regular medications included levothyroxine and omeprazole. There was no relevant family history. On examination, there was a 4mm firm papule present on the right lower eyelid (Figure 1). The clinical differential diagnosis included syringoma, sebaceous hyperplasia, seborrheic keratosis and, less likely, basal cell carcinoma (BCC). The lesion was surgically excised and sent to the pathology laboratory for further analysis.

**Figure 2:**
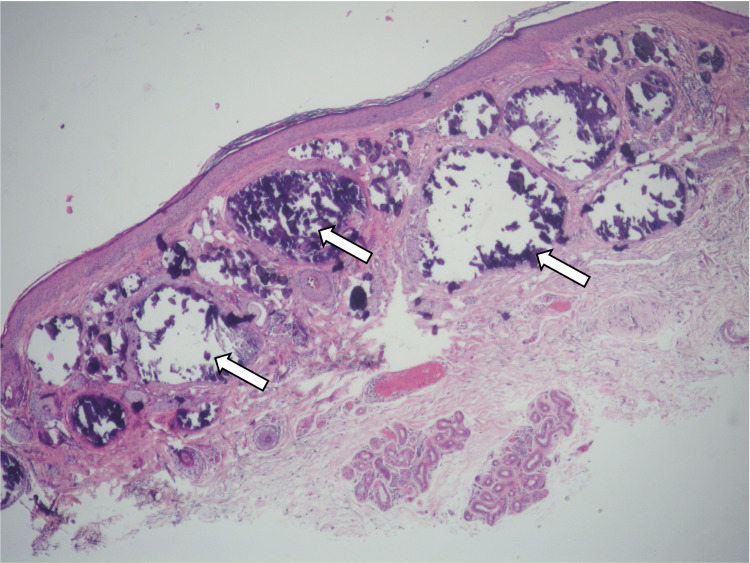
This low-power-view image shows heavily calcified dermal nodules (arrows) with unremarkable overlying epidermis. Taken in context of the whole lesion, the syringomatous component is scanty and almost unnoticeable at this power.

**Figure 3:**
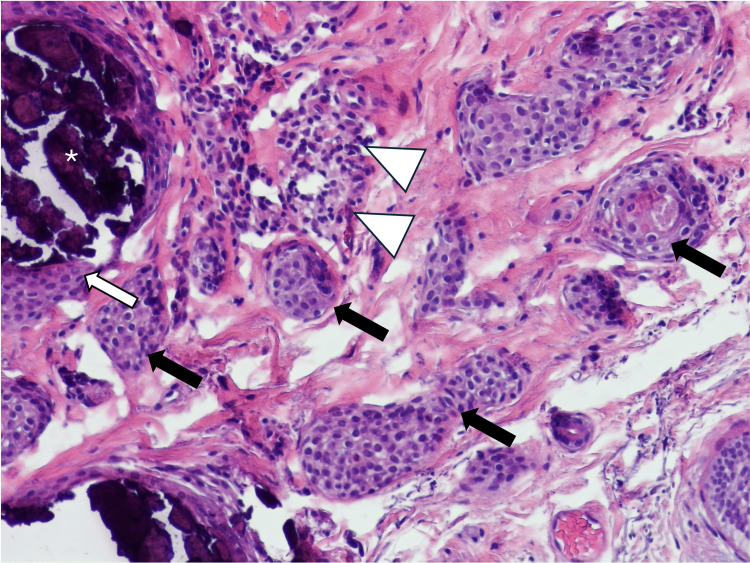
High-power-view shows tear-drop-shaped “syringomatoid” nests (black arrows) immediately adjacent to calcification (white asterisk) located within a dilated sweat duct (white arrow). Note that the ductal cells lining the calcified area show nearly identical appearance to the syringomatoid nests. Focal lymphocytic infiltrate is seen involving a couple of the ductal structures in this image (white triangles) – a finding described in other cases of syringomatoid hyperplasia.

**Figure 4:**
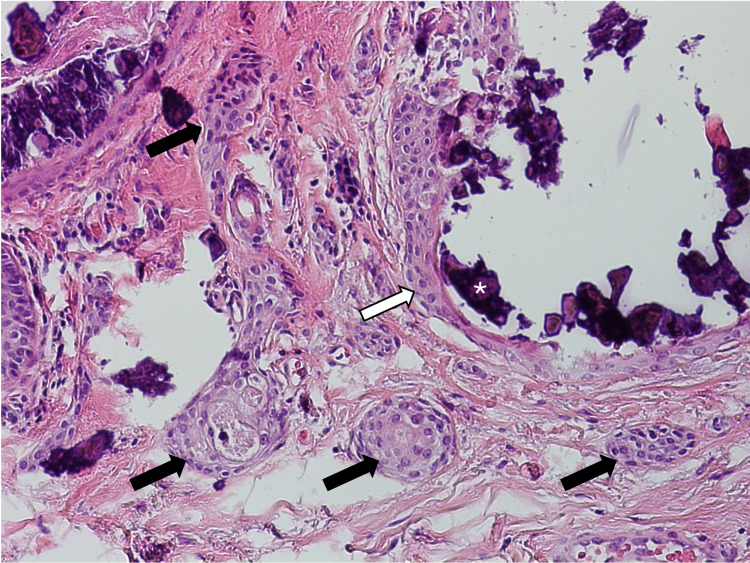
Another high-power view demonstrating calcification (asterisk) occupying a dilated eccrine duct (white arrow) with more typical comma-shaped ducts adjacent (black arrows).

Histology of the lesion showed large, calcified nodules occupying much of the superficial dermis (Figure 2). The overlying epidermis was unremarkable. On higher power, numerous bilayered, comma-shaped ductular nests were discovered adjacent to the calcification in the superficial dermis (Figure 3 and Figure 4). Some of these ducts showed mild infiltration by chronic inflammatory cells. Further inspection noted that the calcified nodules were contained within a bilayered ductular epithelium identical to the ductular components described. No other abnormality was present. The case was signed out as calcinosis cutis with syringomatous changes. The patient did not report any recurrence or further lesions since the excision.

## Discussion

Syringomas are well-recognized benign skin adnexal tumors believed to arise from the acrosyringium [1]. They typically present as small flesh-colored papules on the eyelids [2]. Histologically, they demonstrate bland, bilayered comma-shaped or tadpole eccrine ducts in the superficial dermis [2]. Reactive eccrine ductal proliferations producing similar “syringomatoid” ducts have long been described in the literature [3]. The comma-shaped appearance of the ducts is a product of cross-sectioning of hypercoiled glands [3]. Numerous skin conditions have been implicated as the driving pathology and presumably occur as a consequence of ductal obstruction or direct irritation of the eccrine ducts themselves (see Table 1).

**Table 1:** Lesions described with eccrine ductal proliferation Table credits: Authors' creation. All information obtained is from [3] unless a separate citation is present. SCC: Squamous cell carcinoma; BCC: Basal cell carcinoma

Category	Entity
Epithelial neoplasms	Keratoacanthoma
	SCC
	Superficial BCC
Melanocytic neoplasms	Melanocytic naevi
	Malignant Melanoma
Inflammatory and other dermatoses	Eczema [4]
	Prurigo nodularis [5]
	Lichen planus
	Pityriasis lichenoides chronica
	Acne rosacea
	Scarring and non-scarring alopecia [6]
	Epidermolysis bullosa
	Chondrodermatitis nodularis helicis
	Porphyria cutanea tarda
	Porokeratosis of Mibelli
Other lesions	Trichilemmal cyst
	Fibroma
	Nasal glioma [7]
	Calcinosis cutis [this case]

The cut-off between syringomatoid hyperplasia and syringomas is somewhat subjective. Generally, syringomatoid hyperplasia is less extensive than syringoma and would have some sort of inducing factor, such as a neoplasm or inflammatory insult. Interestingly, some authors believe the variant of syringoma, known as eruptive syringoma, which is characterized by the sudden eruption of multiple syringomas on two or more anatomical parts, is in fact a form of syringomatoid hyperplasia which is hormonally driven [2, 4]. It is possible that syringomatoid hyperplasia and syringoma represent two points on a spectrum of reactive eccrine ductular proliferations. No inciting gene mutation for syringomas has ever been described, further adding weight to this theory.

In our case, we observed an isolated skin lesion on the eyelid, which was composed primarily of calcification (i.e., calcinosis cutis) and possessed a lesser component of syringomatous ducts. Calcinosis cutis is a category of skin lesions characterized by calcium-salt deposition in the soft tissue of the skin and can broadly be considered as dystrophic, metastatic or idiopathic (Table 2). We considered the possibility that the histological findings identified in our case could have represented a syringoma with dystrophic calcification - after all, calcification occurring in syringomas has been described, albeit rarely [8-16]. In general, the degree of calcification in these cases was relatively mild in comparison to what we observed in our case. In our view, the relative scarcity of reports of dystrophic calcification in syringomas, the extensiveness of the calcification, the presence of a solitary rather than multiple lesions on the eyelid and the age of the patient favor a diagnosis of calcinosis cutis-inducing syringomatoid hyperplasia, rather than syringoma. However, the possibility of prominent dystrophic calcification occurring in a syringoma, especially as the lesion was present at a typical site, cannot be entirely excluded.

**Table 2:** Subtypes of calcinosis cutis Table credits: Authors' creation using information from [17].

Subtype of calcinosis cutis	Distinguishing features
Dystrophic calcification	Cutaneous calcium deposition developing in lesional tissue in the setting of normal calcium/phosphate levels
Metastatic calcinosis cutis	Cutaneous calcium deposition in the setting of abnormal calcium and/or phosphate metabolism and no evidence of background lesional skin (i.e., hypercalcemia or hyperphosphatemia)
Idiopathic calcinosis cutis	Cutaneous calcium deposition occurring in the setting of no apparent background skin lesion and no calcium or phosphate metabolic abnormality. Well-known examples include scrotal calcinosis, tumoral calcinosis (periarticular skin) and solitary calcified nodules (face of children)
Iatrogenic calcinosis cutis	Cutaneous calcium deposition related to certain treatments e.g. IV calcium gluconate, subcutaneous para-aminosalicylic acid, blood products, post-transplant
Calciphylaxis	Cutaneous calcium deposition located in the walls of small vessels of the dermis and subcutaneous fat, usually in the setting of end-stage renal disease

To the best of our knowledge, this is the first reported case of syringomatoid hyperplasia with calcinosis cutis, although it should be mentioned that a report by Maroon et al. in 1990 [16] demonstrated calcinosis cutis with multiple syringomas, which in hindsight may have represented syringomatoid hyperplasia rather than syringomas, but this is difficult to conclude without reviewing the case.

## Conclusions

To the best of our knowledge, this is the first reported case of syringomatoid ductal hyperplasia occurring secondary to calcinosis cutis. This was an unusual case, which could have been interpreted in one of two ways. Either explanation is unlikely to have significantly altered the management of the patient, but resurfaces an interesting discussion point about the true nature of syringomas and whether they represent a reactive/hyperplastic phenomenon or are truly a neoplastic lesion. Despite showcasing a rare morphological phenomenon, this case serves to remind that all that is comma-shaped is not unequivocally syringoma. The diagnosis of a syringoma needs to be considered along with the clinical context and careful histological evaluation of background skin (for potential duct-inducing pathologies) before a diagnosis is made.
